# Accurate Estimation of Methemoglobin and Oxygen Saturation in Skin Tissue Using Diffuse Reflectance Spectroscopy and Artificial Intelligence

**DOI:** 10.1002/jbio.202400413

**Published:** 2025-02-26

**Authors:** Isra Sahli, Wesam Bachir, Moustafa Sayem El‐Daher

**Affiliations:** ^1^ Biomedical Photonics Laboratory Higher Institute for Laser Research and Applications, Damascus University Damascus Syria; ^2^ Institute of Metrology and Biomedical Engineering, Faculty of Mechatronics, Warsaw University of Technology Warsaw Poland

**Keywords:** artificial neural networks, diffuse reflectance spectroscopy, methemoglobin, Monte Carlo simulations

## Abstract

In this paper, we present a noninvasive method for the accurate estimation of methemoglobin concentration. The proposed technique incorporates a novel machine learning model using the artificial neural network to detect methemoglobin and oxygen saturation from the diffuse reflectance spectra of skin tissue. Sixty‐six spectra were simulated using a four‐layer tissue model with varying oxygen saturation and methemoglobin concentration. A multifiber probe‐based DRS setup in the visible and near‐infrared wavelength range was used. The best accuracy, with a mean absolute error (MAE) of 0.0392% for the concentration of methemoglobin and 0.0273% for the percentage of oxygen saturation on the created data set, was achieved. Our method was also experimentally verified using DRS spectra collected from human subjects. Consequently, the findings demonstrate the ability of broadband DRS to noninvasively differentiate subtle changes in methemoglobin and hemoglobin levels despite their overlapping spectral features.

## Graphical abstract

Discover a noninvasive method for estimating methemoglobin levels in human tissue with remarkable precision. By leveraging a novel machine learning approach using artificial neural networks, this study showcases the potential of diffuse reflectance spectroscopy (DRS) to accurately detect methemoglobin and oxygen saturation. Experimentally validated on human subjects, the method achieves unprecedented accuracy, paving the way for advanced diagnostics and real‐time health monitoring without the need for invasive procedures.

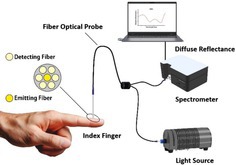

## 1 Introduction

Methemoglobin (MetHb) is a dysfunctional hemoglobin derivative that is formed naturally in human blood at a rate of up to 3% of total hemoglobin [1]. High levels of MetHb concentration may result from congenital enzyme deficiencies, chemicals, or drugs such as local anesthesia [2]. Therefore, the determination of MetHb concentration is significant in diagnosis because it refers to the inability of the blood to transport oxygen throughout the body, leading to tissue hypoxemia [3], myocardial infarction, or even, in the worst‐case scenario, death if it is not treated immediately [4] especially when its concentration is above 60% [5]. Moreover, congenital defects in newborns, infants, and children with cyanosis not associated with hypoxia were detected when their measured MetHb concentration was greater than 10% [6]. The mild conditions can be treated by removing the provoking substance, delivering oxygen, and monitoring, whereas the critical conditions may be treated by providing methylene blue [7]. Therefore, prior knowledge of the MetHb concentration is the most important step in treatment.

There exist many techniques for the determination of MetHb concentration; the gold standard method is blood gas analysis, which is an invasive method and not suitable for continuous monitoring. The co‐oximetry technique can also be used for MetHb measurements; however, it requires intermittent blood drawing, and it has been proven that MetHb concentration increases with time [8] and these devices often have difficulties differentiating between sulfhemoglobin, methylene blue, and MetHb [9]. Gewiß et al. [10] used a pulse oximeter to evaluate MetHb concentration by utilizing more wavelengths from 500 to 1400 nm, but their results were not accurate enough. Likewise, Tang et al. [11] demonstrated their ability to determine the presence of MetHb by studying and representing its distribution inside the phantoms and in the mouse ear using three‐dimensional photoacoustic microscopy, but their technique has not been investigated on human subjects.

On the other hand, other approaches for measuring MetHb have so far been reported, especially those that depend on spectrometric analysis. For example, Cruz‐Landeira et al. [12] determined MetHb saturation percentage and total hemoglobin concentration from the first derivative of the absorption spectrum at 645 nm for blood samples; however, they have not tested the effectiveness of their method in continuous and real‐time measurement in vivo. Bachir and Hamadah [13] extracted tissue oxygen saturation (StO_2_) using the second derivative of the diffuse reflectance spectrum in the visible wavelength range, and their study did not include the effects of all hemoglobin derivatives, particularly MetHb. Moreover, Khatun et al. [14] provided a new methodology to estimate MetHb, oxyhemoglobin, deoxyhemoglobin concentrations, and tissue StO_2_ from diffuse reflectance images reconstructed from RGB images by Wiener estimation. Their work, however, lacks an evaluation of the effect of melanin level on the results; similarly, Park et al. [15] measured blood hemoglobin levels from the RGB eyelid images using spectral super‐resolution spectroscopy. This application needs a large number of tests, including a systematic analysis.

Despite all the previously mentioned techniques, it is virtually impossible to make measurements on humans because many variables differ from one person to another, so there is a pressing need for a new methodology that can tackle this issue. Computational methods can facilitate such investigations, particularly simulation methods using multiple variables that may affect the measurements. For instance, Chatterjee et al. [16] provided a Monolayer Monte Carlo (MC) simulation that simulates light‐skin tissue (dermis layer only) interactions at 660 and 940 nm; they have proven that the optical path depends on blood volume and blood StO_2_, but their studies would have been better if they expanded their investigation to include specialized layers and more variables. Chatterjee et al. [17] implemented the MC simulation on a model that simulates the human index finger, and they relied on the transmittance and reflectance modalities of photoplethysmography (PPG); their analysis showed that the effect of dermis and bones on the PPG signal differs between individuals. Although the variation in blood volume by blood pressure in a tissue volume may affect the results but their study did not discuss it.

Owing to the latest advancements in signal and image processing that have taken place over the past decades along with the complexity of some experimental studies, such as modern studies in tissue optics, it has been necessary to introduce machine learning techniques to algorithms that are intended to estimate vital parameters, especially those that depend on PPG as a noninvasive method [18]. Much of the previous research discussed those methods, like Lakshmi et al. [19], who predicted hemoglobin levels from a PPG signal and its derivatives for pregnant women using the linear regression technique; their results were not satisfactory as there was no correlation between the predicted values and the actual values of hemoglobin. This can be attributed to the small database and the lack of samples containing abnormal values such as anemia. Eight machine learning techniques were tested by Kavsaoʇlu et al. [20] to predict hemoglobin levels from PPG signals and their derivatives; they have proven that the support vector regression (SVR) technique was the best technique according to the R‐squared performance criteria.

Banerjee et al. [21] developed an intelligent method to estimate hemoglobin, bilirubin, and StO_2_ in neonates at the same time; their procedure relies on illuminating the neonatal nail with white light and then acquiring diffuse reflectance spectra that they use to train the artificial neural network (ANN) which includes five hidden layers to increase the accuracy of the results. This method demonstrates the potential to predict the onset of heart disease through the levels of StO_2_ in the blood. In another study by Hoffman et al. [22], they developed a method to detect hypoxemia from blood StO_2_ similar to the pulse oximeter using a smartphone camera and deep learning model (CNN) with 81% sensitivity and 79% specificity. Their results were suitable to the research sample size, which was 6 in total; however, it would be possible to adopt their method as an alternative to the standard pulse oximeter if their research sample was larger and more inclusive (such as containing different skin colors).

In this paper, we propose a new method for accurately predicting MetHb concentration and StO_2_ based on diffuse reflectance spectra obtained through MC simulations of multi‐layered skin tissues. The predictive capabilities of the ANN were validated by applying it to diffuse reflectance spectra collected from the fingers of five healthy volunteers. The novelty of this work lies in addressing the complexities of tissue optics, particularly the influence of chromophores on MetHb levels. By incorporating all relevant chromophores into the MC model and simulations, we achieve a more realistic representation of tissue. To our knowledge, this is the first approach leveraging an ANN for the automatic and accurate estimation of MetHb concentration. Furthermore, this is the first study where MetHb concentration and StO_2_ have been estimated from both simulated and human data in a noninvasive manner. This work also represents the first prediction of MetHb concentration alongside StO_2_ using ANN. Moreover, the known relationship between MetHb and StO2, as established in prior studies, is taken into account in our approach. Since experimental procedures for quantifying MetHb concentration are often challenging without certain assumptions, the development of the ANN‐based algorithm in this paper to predict MetHb and StO_2_ from simulated diffuse reflectance spectra is presented and subsequently validated against human data.

## 2 Materials and Methods

Diffuse reflectance spectroscopy (DRS) has been a promising modality that provides valuable information about the optical properties of biological tissue of interest. The skin tissue is composed of scatterers and absorbers (chromophores) with certain concentrations, the change of which may indicate the presence of an abnormality or malfunction in biological tissue. Moreover, the spectral features of the diffuse reflectance spectrum highly depend on the absorption and scattering coefficients of the tissue. To simulate diffuse reflectance in a skin tissue model, a multilayered MC simulation was employed. The simulation algorithm and the model as well as the developed ANN are described in detail in the following sections.

### 2.1 Monte Carlo Simulation

MC simulation is a statistical method that describes the interaction of photons with turbid tissue; it has given the ability to simulate any tissue regardless of its complex composition and optical properties [23].

In the present work, Monte Carlo Multi Layers (MCML) were implemented after modifying the code to simulate the fibers used in real‐world experiments. More specifically, the illumination fiber is placed at the origin of the cartesian coordinates (*x*, *y*, *z*), and collection fibers are placed just on the *x*‐axis, while in our code, the collection fibers can be placed at any location above the skin surface.

**FIGURE 1:**
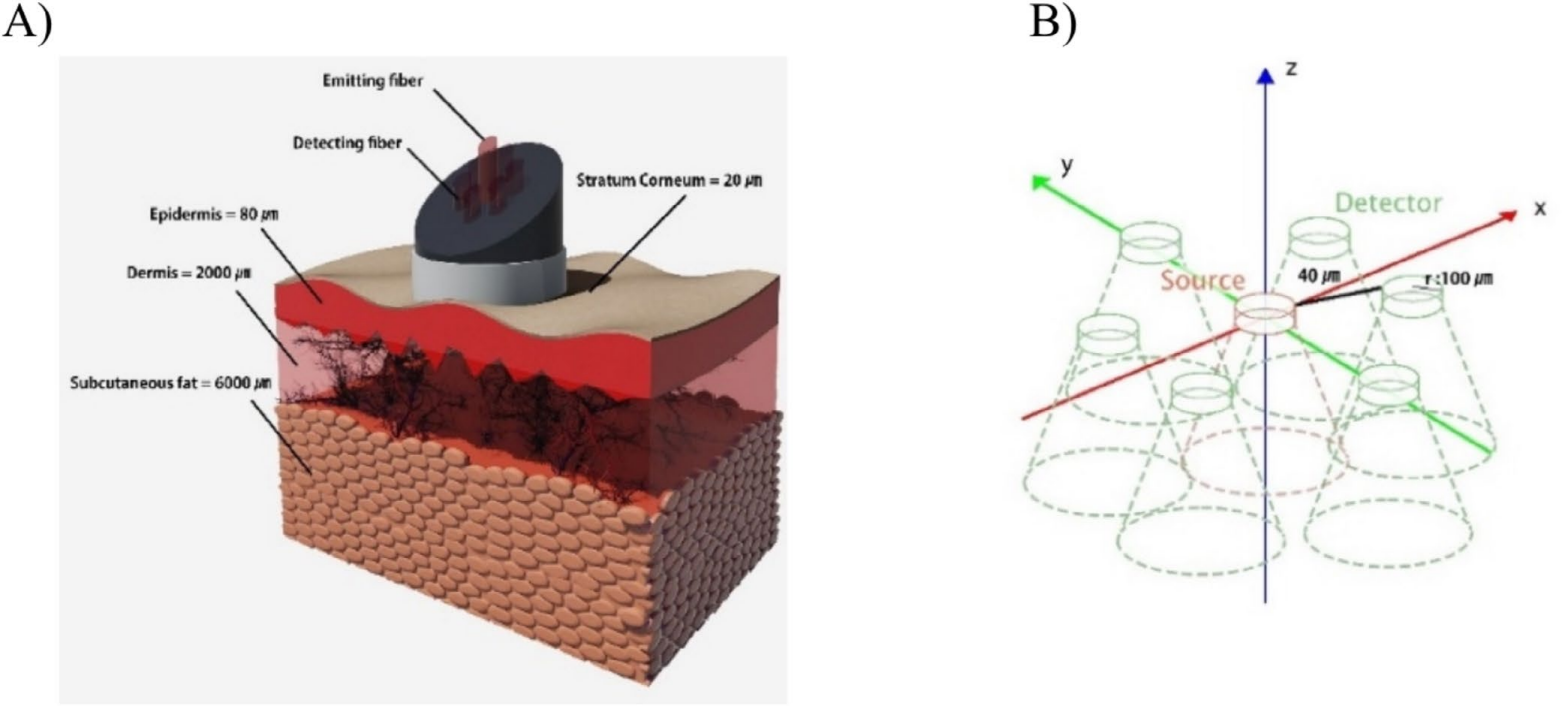
(A) Schematic representation of a four‐layer skin tissue model: the Stratum corneum, Epidermis, Dermis, and Subcutaneous fat layer. (B) The geometry of the MC model is presented in Cartesian coordinates (*x*, *y*, *z*), and the illumination fiber is surrounded by six collection fibers.

We used one illumination fiber with a 100 μm radius and six collection fibers with a 100 μm radius. The collection fibers are placed around the illumination fiber. The distance between the center of the illumination fiber and the center of any of the collection fibers was 240 μm. For the simulation, the voxel size used was $0.005\times 0.005\times 0.005\;\left[{\mathrm{cm}}^{3}\right]$, $10^{6}$ photons were used at each single wavelength in the range of visible and near‐infrared, that is, from 400 to 1000 nm at 2 nm intervals. The geometry of the fiber probe model is shown in Figure 1. The simulation was run with the aid of a computer with a Windows 10 64‐bit operating system, 64 GB RAM, and an Intel(R) Core (TM) i7‐6850K CPU at 3.60 GHz processor, the average computational time of MC simulation for each single spectrum (from 400 to 1000 nm) is 2 h. Reflectance spectra for different values of chromophore concentration and volume fraction can be obtained and it is considered that the diffuse reflection is the sum of all the diffuse reflections calculated by the detecting fibers.

### 2.2 Optical Model of Skin Tissue

The skin tissue model mimics four layers: Stratum corneum, Epidermis, Dermis, and Subcutaneous fat, as shown in Figure 1. Air and bone are the top and the bottom media, respectively. Optical coefficients that describe the skin tissue are related to the number of absorbers and scatterers existing in the layer, and hence, the total absorption coefficient can be expressed by [24].

(1) $$\begin{array}{c} \;\;\mu_{a}=\sum_{i\;}{\mathrm{vf}}_{i}\;\mu_{a\_i}\;\left({\mathrm{cm}}^{-1}\right) \end{array}$$

where ${\mathrm{vf}}_{i}$ is the volume fraction, $\mu_{a\_i}$ is the absorption coefficient of the chromophore (i), and the external layer is the stratum corneum with 20 μm thickness [25]. This layer contains only water, therefore its absorption coefficient given that the volume fraction of water is ${\mathrm{vf}}_{w}$ [26], is

(2) $$\;\mu_{a\_\text{stratum}\_\text{corneum}}\left(\lambda \right)={\mathrm{vf}}_{w}\;\mu_{a\_w}\left(\lambda \right)+\left(1-{\mathrm{vf}}_{w}\right)\;\mu_{a_{\text{baseline}}}\left(\lambda \right)\;\left({\mathrm{cm}}^{-1}\right)$$

Where the water absorption coefficient $\mu_{a\_w}\left(\lambda \right)$ (at a hematocrit of 45%) was taken from tabulated data published in the literature [27], the baseline absorption coefficient $\mu_{a\_\text{baseline}}\left(\lambda \right)$ (the absorption coefficient of the dermal sublayer in the absence of any other chromophore) can be determined by [17, 28]

(3) $$\;\mu_{a_{\text{baseline}}}\left(\lambda \right)=7.84\times 10^{8}\times \lambda^{-3.255}\;\left({\mathrm{cm}}^{-1}\right)$$

The second layer is the epidermis with 80 μm thickness [25]. It contains melanin and water as absorbers [29], melanin absorption coefficient $\mu_{a\_\mathrm{me}}\left(\lambda \right)$ can be calculated by using the following equation [28].

(4) $$\mu_{a\_\mathrm{me}}\left(\lambda \right)=6.6\times 10^{11}\times \lambda^{-3.33}\;\left({\mathrm{cm}}^{-1}\right)$$

The volume fraction of melanin is ${\mathrm{vf}}_{\mathrm{me}}$, thus the total absorption coefficient of the epidermis layer is given by the following equation

(5) $$\begin{array}{c} \mu_{a\_\text{epidermis}}\left(\lambda \right)={\mathrm{vf}}_{w}\;\mu_{a\_w}\left(\lambda \right)+{\mathrm{vf}}_{\mathrm{me}}\;\mu_{a\_\mathrm{me}}\left(\lambda \right)\\ \;+\left(1-\left({\mathrm{vf}}_{\mathrm{me}}+{\mathrm{vf}}_{w}\right)\right)\;\mu_{a\_\text{baseline}}\left(\lambda \right)\;\left({\mathrm{cm}}^{-1}\right) \end{array}$$

For the third layer (i.e., the dermis), the thickness is 2000 μm [25]. Likewise, this layer contains many absorbers, particularly oxyhemoglobin, deoxyhemoglobin, MetHb, water, bilirubin, and beta‐carotene [24, 25, 30].

Based on the assumption that the volume fraction of the blood is ${\mathrm{vf}}_{\mathrm{bl}}$, the total dermis absorption coefficient can be expressed as

(6) $$\begin{array}{c} \mu_{a\_\text{dermis}}\left(\lambda \right)={\mathrm{vf}}_{\mathrm{bl}}\;\mu_{a\_\mathrm{bl}}\left(\lambda \right)+{\mathrm{vf}}_{w}\;\mu_{a\_w}\left(\lambda \right)+2.3\;C_{\mathrm{bi}}\varepsilon_{\mathrm{bi}}\left(\lambda \right)\\ \;+\mu_{a\_\mathrm{bet}}\left(\lambda \right)+\left(1-\left({\mathrm{vf}}_{\mathrm{bl}}+{\mathrm{vf}}_{w}\right)\right)\mu_{a_{\text{baseline}}}\left(\lambda \right) \end{array}$$

$\mu_{a\_\mathrm{bl}}\left(\lambda \right)$ is the absorption coefficient of blood can be expressed as [17].

(7) $$\mu_{a\_\mathrm{bl}}\left(\lambda \right)=\mu_{a\_A\_\mathrm{bl}}\left(\lambda \right)+\mu_{a\_V\_\mathrm{bl}}\left(\lambda \right)$$

where $\mu_{a\_A\_\mathrm{bl}}\left(\lambda \right)$ and $\mu_{a\_V\_\mathrm{bl}}\left(\lambda \right)$ are the absorption coefficients of the arterial and venous blood, respectively, which can be written as [17].

(8) $$\mu_{a\_A\_\mathrm{bl}}\left(\lambda \right)=\mathrm{Sa}O_{2}\;\mu_{a\_\mathrm{oxy}}\left(\lambda \right)+\left(1-\mathrm{Sa}O_{2}\right)\left(\mu_{a\_\text{deoxy}}\left(\lambda \right)+\mu_{a\_\mathrm{met}}\left(\lambda \right)\right)$$

(9) $$\mu_{a\_V\_\mathrm{bl}}\left(\lambda \right)=\mathrm{Sv}O_{2}\;\mu_{a\_\mathrm{oxy}}\left(\lambda \right)+\left(1-\mathrm{Sv}O_{2}\right)\left(\mu_{a\_\text{deoxy}}\left(\lambda \right)+\mu_{a\_\mathrm{met}}\left(\lambda \right)\right)$$

where $\mathrm{Sa}O_{2}$ and $\mathrm{Sv}O_{2}$ are the arterial and venous oxygen saturation, respectively. Six levels of $\mathrm{Sa}O_{2}$ were considered (98%, 97%, 85%, 70%, 56%, and 43%), while $\mathrm{Sv}O_{2}$ was considered 10% lower than the $\mathrm{Sa}O_{2}$ [17], $\mu_{a\_\mathrm{oxy}}\left(\lambda \right)$, $\mu_{a\_\text{deoxy}}\left(\lambda \right)$, and $\mu_{a\_\mathrm{met}}\left(\lambda \right)$ are the absorption coefficients of oxyhemoglobin, deoxyhemoglobin, and MetHb, respectively. These absorption coefficients can be determined using the following equation [24, 29]:

(10) $$\mu_{a\_\mathrm{Hb}}\left(\lambda \right)=2.303\;\frac{c_{\mathrm{Hb}}}{P_{\mathrm{MHb}}}\;\varepsilon_{\mathrm{Hb}}\left(\lambda \right)$$

The extinction coefficient for hemoglobin derivatives $\varepsilon_{\mathrm{Hb}}\left(\lambda \right)$ (oxyhemoglobin and deoxyhemoglobin), bilirubin $\varepsilon_{\mathrm{bi}}\left(\lambda \right)$, beta‐carotene absorption coefficient $\mu_{a\_\mathrm{bet}}\left(\lambda \right)$ was taken from tabulated data given by Prahl [31, 32, 33], while the MetHb extinction coefficient was taken from tabulated data published in the literature [34]. It must be noted here that $P_{\mathrm{MHb}}$ is the gram molecular weight of hemoglobin equal to 64 500 (g/mol), whereas the concentration of bilirubin $C_{\mathrm{bi}}$ was $1.71\times 10^{-6}$ (moles) [24]. In this study, the percentage of MetHb concentration varied from 0% to 100% with an increment of 10% of the total hemoglobin concentration, which was 150 (g/L) [31]. Thus, the concentration of hemoglobin derivatives $c_{\mathrm{Hb}}$ was calculated by the following expressions:

(11) $$c_{\mathrm{met}}=\text{percentage of MetHb concentration}\times 150\;\left(g/L\right)$$

(12) $$c_{\mathrm{oxy},\text{deoxy}}=150-c_{\mathrm{met}}\;\left(g/L\right)$$

The subcutaneous fat layer, which is the last layer with a thickness of 6000 μm [25, 26], it contains water, fat, and blood as absorbers [24], so the following equation determines the subcutaneous fat absorption coefficient.

(13) $$\begin{array}{c} \mu_{a\_\mathrm{sub}\_\mathrm{fat}}\left(\lambda \right)={\mathrm{vf}}_{\mathrm{bl}}\;\mu_{a\_\mathrm{bl}}\left(\lambda \right)+{\mathrm{vf}}_{w}\;\mu_{a\_w}\left(\lambda \right)+{\mathrm{vf}}_{f}\;\mu_{a\_f}\left(\lambda \right)\\ \;+\left(1-\left({\mathrm{vf}}_{\mathrm{bl}}+{\mathrm{vf}}_{w}+{\mathrm{vf}}_{f}\right)\right)\;\mu_{a\_\text{baseline}}\left(\lambda \right) \end{array}$$

$\mu_{a\_f}\left(\lambda \right)$ is the absorption coefficient of fat (taken from tabulated data given by van Veen et al. [35]), the parameters used in our model are listed in Table 1.

**TABLE 1:** Parameters used for our model of the skin tissue.

The layer	${\mathrm{vf}}_{w}$ (%)	${\mathrm{vf}}_{\mathrm{me}}$ (%)	${\mathrm{vf}}_{\mathrm{bl}}$ (%)	${\mathrm{vf}}_{f}$ (%)
Stratum corneum	5	0	0	0
Epidermis	20	2.5	0	0
Dermis	65	0	0.2	0
Subcutaneous fat	15	0	7	60

*Note*: the parameters were adapted from the literature [17, 24, 25].

The dermal layer contains a single scatterer of collagen fibers, so the reduced scattering coefficient $\;{\mu_{s}}^{\prime }\left(\lambda \right)$ and scattering coefficient $\mu_{s}\left(\lambda \right)$ were calculated based on [24, 25], where the anisotropy factor (g) was set as constant with a value of 0.86 [24, 26, 29]. The Parameters specifying the reduced scattering coefficient of the skin layers are listed in Table 2, and all the equations were evaluated using MATLAB (MathWorks Inc. USA, version 2017b).

**TABLE 2:** The parameters specifying the reduced scattering coefficient of the skin layers [24].

Layer	$a^{\prime }\left({\mathrm{cm}}^{-1}\right)$	$f_{\mathrm{Ray}}$	$b_{\mathrm{Mie}}$
Epidermis	66.7	0.29	0.689
Dermis	43.6	0.41	0.562
Subcutaneous fat	34.2	0.26	0.567

The refractive index of the stratum corneum and the subcutaneous fat was 1.55 and 1.44, respectively, while for the epidermis and dermis, it was calculated based on [25] for each wavelength used in this study.

### 2.3 Study Design and Experimental Setup

To assess the performance of the proposed method, diffuse reflectance spectra were collected from the index fingers of five subjects, aged between 25 and 60 years. Diffuse reflectance measurements were collected from five volunteering subjects, all of whom were nonsmokers, free from chronic diseases, and not taking any medications. Before data collection, the measurement procedure was explained to the participants. This study was approved by the Institutional Ethics Committee of Damascus University, and informed consent was obtained from all volunteers. This study was conducted under the principles of the Declaration of Helsinki.

**FIGURE 2:**
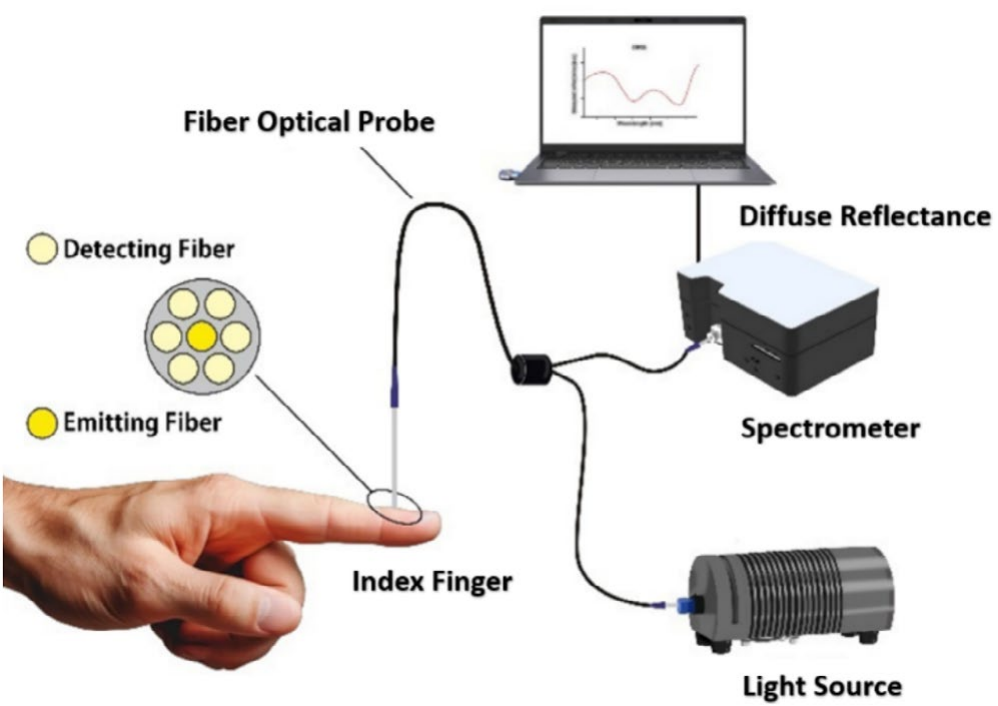
Schematic overview of the optical setup used for measuring diffuse reflectance.

The spectra were acquired using a compact, portable spectrometer (USB4000 FL, Ocean Optics Inc., Dunedin, FL, USA), with the finger tissue illuminated by a broadband light source covering a range of 400–2000 nm (HL‐2000‐FHSA‐HP, Ocean Optics Inc., Dunedin, FL, USA). A bifurcated fiber‐optic probe (EOS‐A1218332, VIS/NIR, Ocean Optics Inc.) was employed to transmit light to the tissue and collect the diffuse reflected light for analysis. The probe consists of six collection fibers arranged around a central illumination fiber, with a fiber diameter of 200 μm and a collection–illumination separation of 240 μm (center‐to‐center), as depicted in Figure 2.

The spectrometer was interfaced with a computer for spectra acquisition and control using proprietary software (Ocean Optics Inc., Dunedin, FL, USA). Then, the collected spectra were processed and analyzed using MATLAB.

In addition to collecting diffuse reflectance spectra, oxygen saturation levels were measured immediately after using a pulse oximeter sensor (AFE4490SPO2EVM, Texas Instruments, USA). Estimated MetHb levels were confirmed via blood tests.

To predict the MetHb and StO_2_ levels of the subjects, an ANN was developed and applied. The collected spectra were preprocessed, including smoothing and feature extraction, before being input into the proposed ANN. The predicted values were then compared with actual values obtained from laboratory analyses and pulse oximetry measurements to assess accuracy.

### 2.4 Signal Processing and Features Extracted

In most cases, preprocessing techniques to reduce noise are crucial for further processing and feature extraction. Smoothing the spectra is one of the widely used approaches for suppressing unwanted high frequencies. More specifically, the Savitzky–Golay smoothing filters, also known as polynomial smoothing or least‐squares smoothing filters, can better preserve the high‐frequency content of the desired spectra [36]. After smoothing the spectra, 12 significant and discriminant features were extracted using MATLAB 2017b (MathWorks Inc. Natick, MA, USA). The features are as follows:

1. Peak analysis: It is important to analyze the peaks that appear in the spectra because they may be an indication of an important parameter. For example, the height and width of the peaks may vary when the concentration of a particular chromophore differs in a sample [18]. Five features were extracted using this method. These include peak amplitude, location of the peak (wavelength), width in half‐height, prominence, and width in half‐prominence.
2. Power of a signal: The power of a signal is the sum of the absolute squares of its time‐domain samples divided by the signal length, or, equivalently, the square of its RMS (root mean square) level [37]. It should be noted here that when the samples are expressed in terms of wavelengths, the wavelength data are transformed into a form that can be interpreted as a frequency domain, with diffuse reflectance serving as the signal's amplitude. Since the values are derived from an MC simulation rather than being mathematically calculated, the power function is still valid and can be used without modification. However, for clarity, we have replaced the time‐domain samples with wavelength values.
3. Kaiser–Teager energy (KTE): It is a useful tool for analyzing signal components where statistical properties such as skewness, variance, kurtosis, mean, standard deviation (SD), and RMS of this energy profile can be calculated [38]. It is given by the following expression in terms of wavelength [39]:

(14) $$\emptyset \left[x\left(n\right)\right]=x^{2}\;\left(n\right)-x\left(n-1\right)x\left(n+1\right)$$

where *n* is the wavelength.

The statistical features used in this work are skewness, variance, kurtosis, mean, root‐mean‐square level, and SD.

Feature selection based on correlation (CFS) is employed to find the optimal feature set. Correlation is a criterion used to measure whether a feature is relevant to other features in the data set and relevant to outputs or not. In other words, the best features should have low linear relations with other features and high linear relations with labels, yielding better performance in terms of accuracy [20]; as a result, by using this method, the features decreased from 12 to 6 features.

### 2.5 ANN

In this work, an ANN was developed to predict the MetHb concentration and $\mathrm{Sa}O_{2}$. It consists of layers of artificial neurons as an input and output of the network [18], where the input layer consists of neurons which were the features mentioned earlier. We built four ANNs to investigate the best model; the first network was built using all of the features that were acquired from the previous step, the second model used just the peak analysis features, the third model used the power of a Signal and KTE features and the last model using features selection based on correlation; thus, the number of neurons in the input layer was 12, 5, 7, 6 respectively, while the output layer consists of two neurons representing the MetHb concentration and $\mathrm{Sa}O_{2}$, and 10 neurons were in the hidden layer.

In this work, conventional validation was performed. 80% of the data were used for training, and the remaining 20% of the data were used for testing. The algorithm was run on a computer with a Windows 10, 64‐bit operating system, 8 GB RAM, and an Intel(R) Core (TM) i5‐7200U CPU @2.50GHz 2.71 GHz processor. The simulation environment was MATLAB 2017b.

Different parameters were used for evaluating the performance of the constructed ANN. The performance‐evaluating criteria to test the performance of developed ANN are the mean absolute error (MAE), mean absolute percentage error (MAPE), mean square error (MSE), root mean square error (RMSE), *R* ^2^ (coefficient of determination). If we consider $y_{i}$ to be the actual values of MetHb concentration and $\mathrm{Sa}O_{2}$, ${\hat{y}}_{i}$ to be the predicted values of MetHb concentration and $\mathrm{Sa}O_{2}$ by the model for the *i*th sample, then the performance‐evaluating criteria are calculated as [19, 20, 40]:

(15) $$\mathrm{MAE}=\frac{1}{n}\;\sum_{i=1}^{n}\left|\left(y_{i}-{\hat{y}}_{i}\right)\right|$$

(16) $$\text{MAPE}=\frac{1}{n}\;\sum_{i=1}^{n}\left|\;\frac{y_{i}-{\hat{y}}_{i}}{y_{i}}\;\right|$$

(17) $$\mathrm{MSE}=\frac{1}{n}\;\sum_{i=1}^{n}{\left(y_{i}-{\hat{y}}_{i}\right)}^{2}$$

(18) $$\text{RMSE}=\sqrt{\mathrm{MSE}\;}$$

(19) $$\;R^{2}\left(y,\hat{y}\right)=1-\frac{\sum_{i=1}^{n}{\left(y_{i}-{\hat{y}}_{i}\right)}^{2}}{\sum_{i=1}^{n}{\left(y_{i}-\bar{y}\right)}^{2}}$$

where *n* represents the number of samples, $\bar{y}$ the average of the actual values is shown in the formula:

(20) $$\;\bar{y}=\frac{1}{n}\;\sum_{i=1}^{n}y_{i}$$

To produce the relationship between the actual and predicted values, we used a regression plotting function provided by MATLAB. This function generates a linear regression plot that compares the actual target values (ground truth) with the predicted values produced by the ANN.

The regression plot displays the network's output on the *y*‐axis against the corresponding target values on the *x*‐axis. A well‐trained network will yield a regression line that closely aligns with the diagonal of the plot, intersecting the bottom‐left and top‐right corners.

**FIGURE 3:**
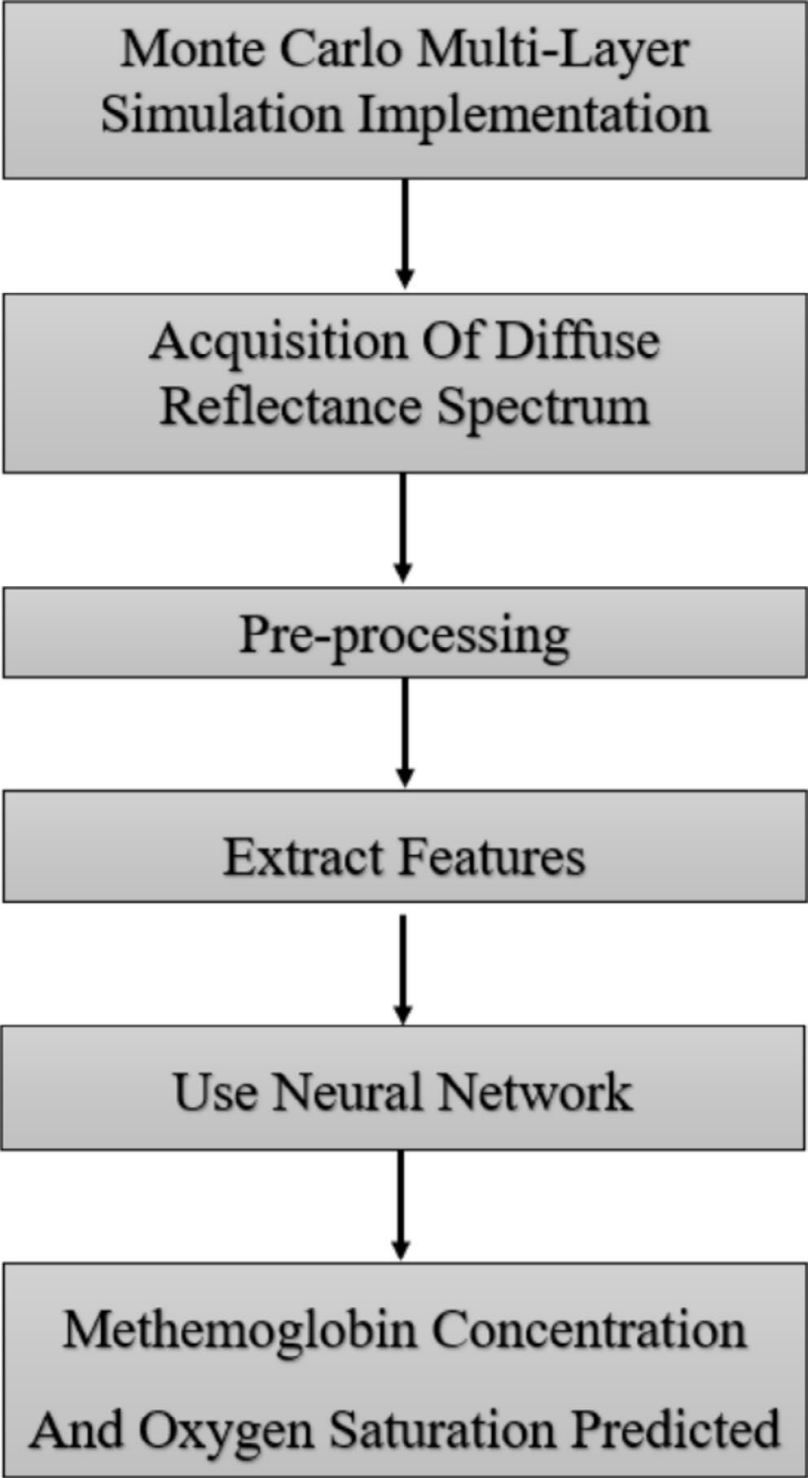
Flowchart of the procedure for estimation of MetHb concentration and $\mathrm{Sa}O_{2}$.

The flow chart in Figure 3 outlines the methodological steps taken in this study for the estimation of MetHb concentration and $\mathrm{Sa}O_{2}$ in skin tissue. The flow chart starts with simulating the light‐skin model using an MCML algorithm. As a result, diffuse reflectance spectra were created. Then, the collected spectra were preprocessed to extract key features that can be employed for constructing the ANN. Finally, the ANN was applied, and the MetHb concentration and $\mathrm{Sa}O_{2}$ levels were determined.

## 3 Results

A total of 66 diffuse reflectance spectra were created and processed, then the signal processing techniques were applied to extract the potential features required for the developed ANN. This is followed by evaluating the ANN performance in predicting the MetHb concentration and $\mathrm{Sa}O_{2}$ levels. Detailed results and findings regarding the procedures mentioned earlier are given in the following subsections.

### 3.1 Diffuse Reflectance Spectra

**FIGURE 4:**
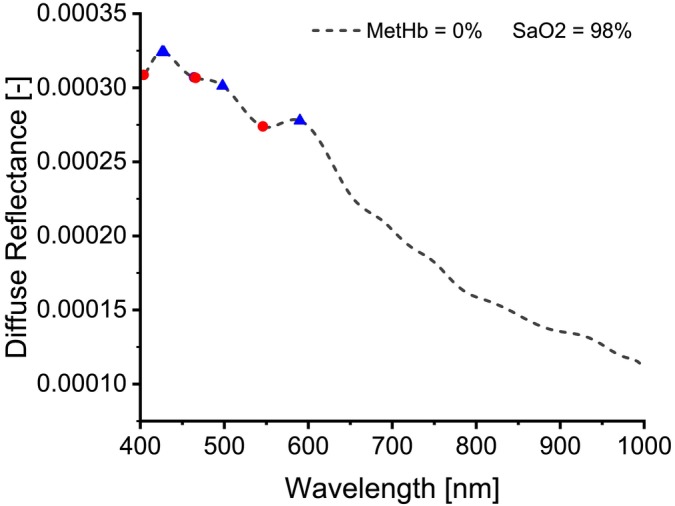
A diffuse reflectance spectrum at 98% $\mathrm{Sa}O_{2}$ and 0% MetHb concentration. Distinctive peaks marked with blue triangles and distinctive valleys marked with red dots can be seen in the visible range of the spectrum.

**FIGURE 5:**
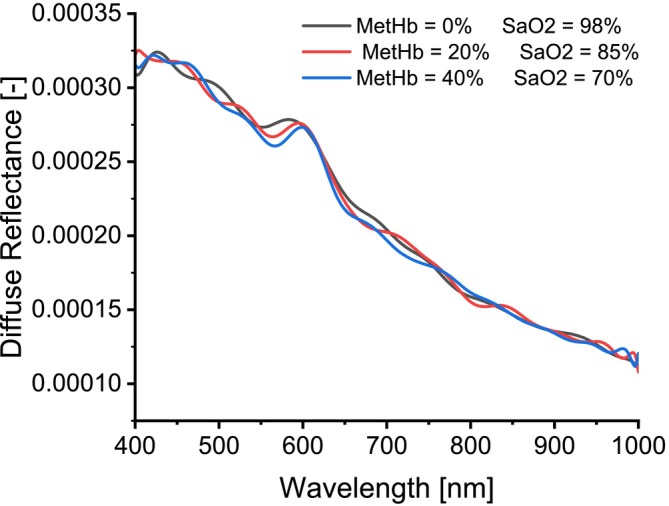
Diffuse reflectance spectra for different MetHb concentrations and $\mathrm{Sa}O_{2}$ levels.

To study the effect of MetHb concentration on the simulated spectra, diffuse reflectance spectra were generated for the skin tissue model using MCML simulation by fixing all the optical properties of the layers except for the absorption coefficient of the dermis and subcutaneous fat layer, which was changed by varying the concentration of MetHb and $\mathrm{Sa}O_{2}$ levels. The MetHb concentration varied from 0% to 100% of the total hemoglobin with a 10% increment. The $\mathrm{Sa}O_{2}$ levels were also varied (98%, 97%, 85%, 70%, 56%, and 43%). As a result, 66 diffuse reflectance spectra were created, but only some of them will be displayed to demonstrate the findings. Figure 4 shows a Diffuse reflectance spectrum at 98% $\mathrm{Sa}O_{2}$ and 0% MetHb concentration, while Figure 5 shows the diffuse reflectance spectra for different MetHb concentrations and $\mathrm{Sa}O_{2}$ levels.

### 3.2 Oxygen Saturation Level vs. Diffuse Reflectance Spectra

**FIGURE 6:**
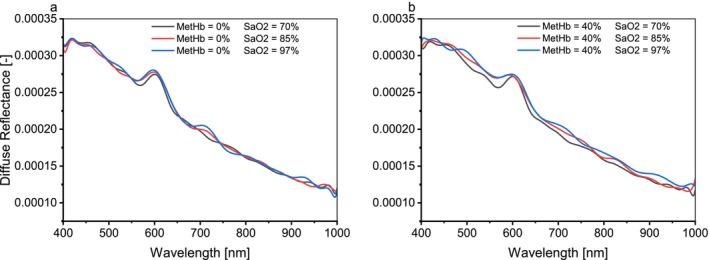
Diffuse reflectance spectra for different values for $\mathrm{Sa}O_{2}$ at MetHb concentration. (a) 0% and (b) 40%.

To quantify the relationship between diffuse reflectance spectra and $\mathrm{Sa}O_{2}$, MCML simulations were conducted multiple times across various $\mathrm{Sa}O_{2}$ levels (98%, 97%, 85%, 70%, 56%, and 43%), while the MetHb concentration varied from 0% to 100% of total hemoglobin in 10% increments. Figure 6 illustrates the diffuse reflectance spectra corresponding to different $\mathrm{Sa}O_{2}$ levels, with MetHb concentrations fixed at 0% and 40%.

### 3.3 Effect of Increased MetHb Concentration on Diffuse Reflectance Spectra

To study the effect of MetHb concentration alone on the diffuse reflectance spectra, attention has been paid to experiments in which the MetHb concentration was increased from 0% to 100% of total hemoglobin in 10% increments at constant $\mathrm{Sa}O_{2}$ levels.

**FIGURE 7:**
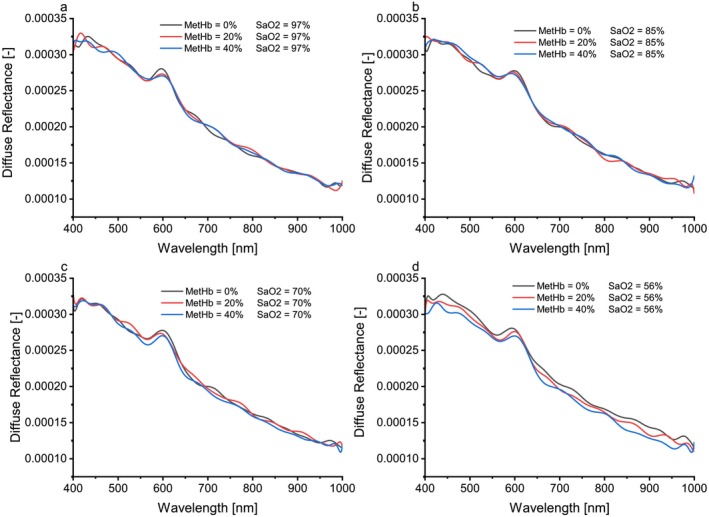
Diffuse reflectance spectra for different values for MetHb concentration and for $\mathrm{Sa}O_{2}$ levels at (a)97%, (b) 85%, (c) 70%, and (d) 56%.

Figure 7 shows the diffuse reflectance spectra at $\mathrm{Sa}O_{2}$ levels (97%, 85%, 70%, and 56%) and MetHb concentrations of 0%, 20%, and 40% of total hemoglobin.

### 3.4 ANN

**FIGURE 8:**
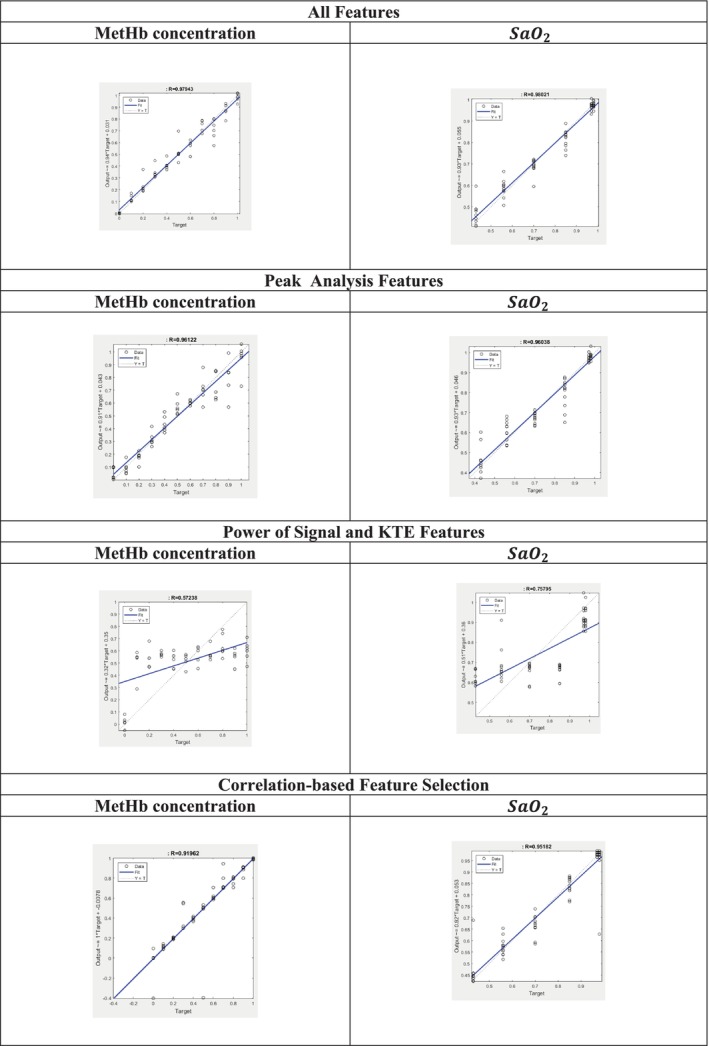
The relationships between the target (the actual values) and predicted values for MetHb concentration and $\mathrm{Sa}O_{2}$.

After applying the developed ANN, it was possible to predict the value of MetHb concentration and $\mathrm{Sa}O_{2}$ from the acquired spectra, the ANN models were trained and tested to evaluate their performance with all features, peak analysis, the power of the signal, KTE features, and feature selection based on correlation. Table 3 provides performance‐evaluating criteria. In addition, the relationships between the target (the actual values) and predicted values of MetHb and $\mathrm{Sa}O_{2}$ can be seen in Figure 8.

**TABLE 3:** Performance‐evaluating criteria for the ANN.

	Performance‐evaluating criteria					
		MAE	MAPE	MSE	RMSE	$R^{2}$
All features	MetHb concentration	0.0392	2.9696	0.0092	0.0959	0.9588
	$\mathrm{Sa}O_{2}$	0.0273	0.0439	0.0068	0.0825	0.9596
Peak analysis features	MetHb concentration	0.0589	5.1107	0.0226	0.1504	0.9235
	$\mathrm{Sa}O_{2}$	0.0374	0.0599	0.0182	0.1352	0.9220
Power of signal and KTE features	MetHb concentration	0.2089	3.6528	0.0670	0.2589	0.3267
	$\mathrm{Sa}O_{2}$	0.1123	0.1793	0.0184	0.1357	0.5668
Correlation‐based feature selection	MetHb concentration	0.0469	7.6153	0.0521	0.2283	0.8163
	$\mathrm{Sa}O_{2}$	0.0314	0.0473	0.0378	0.1946	0.9049

### 3.5 Human Diffuse Reflectance Spectra

**FIGURE 9:**
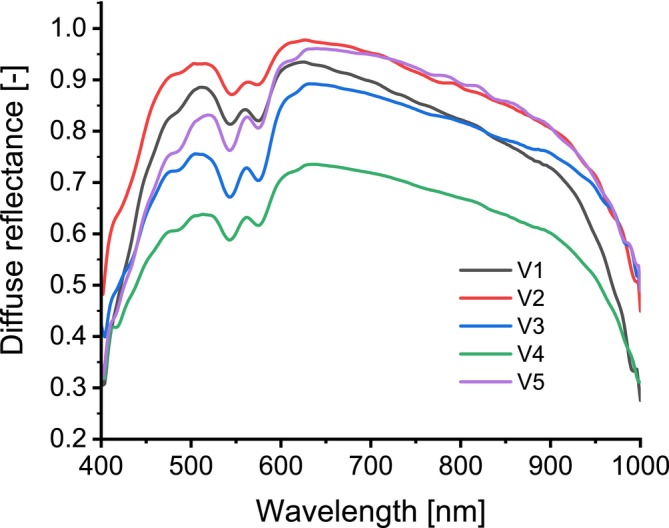
Diffuse reflectance spectra for volunteers.

Diffuse reflectance spectra were collected from five subjects. $\mathrm{Sa}O_{2}$ levels were measured using pulse oximeter and then followed by the analysis of MetHb concentrations. The diffuse reflectance spectra were subsequently smoothed using MATLAB, and the results are presented in Figure 9. Table 4 compares the measured (laboratory blood test) values of $\mathrm{Sa}O_{2}$ and MetHb with the values predicted by the ANN model.

**TABLE 4:** Measured and predicted parameters of volunteers.

Subject	Age	Gender	Measured oxygen saturation (%)	Predicted oxygen saturation (%)	Measured MetHb (%)	Predicted MetHb (%)
1	25	F	97	98.66	1	0.62
2	27	F	97	99.00	1.2	0.75
3	28	M	98	94.42	0.45	2.96
4	41	F	95	98.74	0.61	1.03
5	52	M	96	96.23	0.47	1.04

## 4 Discussion

In this study, we introduced a novel approach to predict MetHb concentration and SaO_2_ levels. The method was applied to diffuse reflectance spectra generated using MCML simulations and obtained from volunteers. Subsequently, signal processing and ANN techniques were utilized to determine MetHb concentration and SaO_2_ levels. To our knowledge, this is the first study to estimate MetHb concentration by analyzing diffuse reflectance spectra combined with artificial intelligence methods.

Examining the diffuse reflectance spectrum at 98% SaO_2_ and 0% MetHb concentration, as shown in Figure 4, reveals that this spectrum resembles those measured from the skin of a healthy human finger. This similarity arises because the simulation reflects the diffuse reflectance of healthy skin tissue with MetHb concentrations ranging from 0% to 3% and SaO_2_ levels exceeding 95%.

Notably, three valleys and three peaks are observed in the visible wavelength range of 400–600 nm. Additionally, a gradual decline in diffuse reflectance is evident toward the end of the visible and near‐infrared range. While consistent with previous findings [41], our spectrum indicates a shift, with the first valley located at 405 nm, and the second and third valleys between 450 and 550 nm. The third peak appears at approximately 585 nm. These spectral shifts may be attributed to differences between measured spectra from finger skin and simulation results. Moreover, In the MC simulation, the light intensity was assumed to be constant across all wavelengths. However, in the real experiments, a white light source was used, and its intensity varies with wavelength. This difference likely contributes to the observed discrepancies between the simulated and real spectra. Hence, incorporating the wavelength‐dependent intensity of the light source into the simulation could produce spectra more closely aligned with the experimental data. We will address this limitation in future work to further refine the simulation model.

To assess the impact of increasing MetHb concentration on diffuse reflectance spectra, MC simulations were performed across various MetHb concentrations and SaO_2_ levels. Spectral analysis revealed that higher MetHb concentrations, corresponding to lower oxygen levels, resulted in a consistent spectral shift to the right, particularly pronounced at 40% MetHb concentration, as shown in Figure 5. In the wavelength range of 400–545 nm, overlapping spectra limited the ability to discern the effect of increased MetHb concentration. However, from 545 to 700 nm, increased MetHb concentration and reduced SaO_2_ levels led to decreased diffuse reflectance, consistent with the absorbing properties of MetHb. Beyond 700 nm, random fluctuations were observed, and spectra were not distinguishable.

Further simulations investigated the effect of decreased SaO_2_ levels at a fixed MetHb concentration of 0%. Figure 6a demonstrates that in the range of 400–545 nm, the impact of reduced SaO_2_ on diffuse reflectance spectra was unclear, whereas between 545 and 655 nm, reduced SaO_2_ levels corresponded to a clear decrease in diffuse reflectance. These findings align with prior work by Can et al. [42], who examined blood bag spectra to estimate hemoglobin concentration. However, differences in spectral shapes are attributable to variations in optical properties between simulated skin tissue layers, incorporating multiple chromophores and scatterers, and blood bags containing only oxyhemoglobin and deoxyhemoglobin.

Simulations at pathological MetHb concentrations verified the relationship between low SaO_2_ and reduced diffuse reflectance under high MetHb conditions (e.g., 40%). As illustrated in Figure 6b, within the 545–655 nm range, diffuse reflectance spectra consistently decreased with declining SaO_2_ levels. Notably, at 70% SaO_2_, the reflectance spectrum was unexpectedly lower, likely due to the combined effects of high MetHb concentration and low oxygenation.

To isolate the effect of MetHb concentration on spectra, SaO_2_ was fixed while MetHb increased from 0% to 100%. As shown in Figure 7a, at 97% oxygen content, diffuse reflectance increased between 545 and 580 nm with rising MetHb, while it decreased between 580 and 620 nm. These results are consistent with those of Delgado et al. [29], who observed reduced diffuse reflectance with increased chromophore concentration. However, outside this specific range, random spectral variations were noted, likely due to the relationship between MetHb and SaO_2_ [14]. Since MetHb cannot bind oxygen, abnormal MetHb levels (above 3%) are typically accompanied by low oxygenation; hence, the result obtained remains correct at an oxygen level of 85, as shown in Figure 7b.

At lower SaO_2_ levels (Figure 7c,d), the increase in MetHb concentration resulted in a regular decrease in diffuse reflectance, particularly evident at a SaO_2_ level of 56%. These findings confirm the anticipated inverse relationship between MetHb concentration, oxygenation, and reflectance.

ANN performance metrics indicated that networks trained with features extracted from diffuse reflectance spectra produced predictions with minimal errors, reflecting high accuracy. When all features were used, the ANN achieved *R* ^2^ values of 0.9588 and 0.9596 for MetHb concentration and SaO_2_ predictions, respectively. Models using only subsets of features (e.g., power of signal and KTE features, or correlation‐based features) showed lower accuracy. The inclusion of all features yielded the lowest prediction errors (MAPE: 2.97% for MetHb concentration, 0.044% for SaO_2_) and outperformed existing devices like the Masimo device, whose relative error ranges from 1% to 15%.

As shown in Figure 8, predictions using the ANN trained on all features were satisfactory, even when compared to models trained on peak analysis or correlation‐based features. However, models using the power of signal and KTE features were less effective, likely due to the lack of significant spectral changes outside the 545–655 nm range. While peak analysis features captured certain trends, their limited scope rendered them insufficient for robust predictions, particularly at constant SaO_2_ levels with varying MetHb concentrations (e.g., Figure 7b).

While the changes between the simulated spectra were small and, in some cases, overlapping, they were sufficient for the ANN to detect and differentiate between them. The ANN utilized a set of features, including those derived from peak analysis, which exhibited slight variations across the spectra. These subtle differences enabled the network to distinguish between the spectra effectively. The developed ANN was then validated using diffuse reflectance spectra obtained from five volunteers (Figure 9). The spectra exhibited variations attributable to differences in hemoglobin concentration, skin thickness, and other individual factors. Predictions for MetHb and SaO_2_ were consistent with actual values, with all predicted MetHb concentrations within the normal range (< 3%).

It is worth noting that changes in optical properties, such as the reduced scattering coefficient, can influence the results obtained in this study. However, the focus of our study was to investigate the effect of variations in the absorption coefficient, specifically related to changes in MetHb concentration and SaO_2_ levels. As such, we concentrated solely on the impact of the absorption coefficient on the diffuse reflectance spectrum. While we recognize the potential limitation of neglecting the scattering effect in this work, we plan to explore this aspect in future studies, particularly how variations in the scattering coefficient may interact with changes in absorption to affect the diffuse reflectance spectrum.

In summary, the proposed ANN, trained on comprehensive spectral features, demonstrates strong potential for accurately predicting MetHb concentration and SaO_2_. This work could pave the way for future advancements in healthcare technologies by incorporating spectra from various body locations to account for factors such as tissue oxygenation, skin color, and scattering effects.

## 5 Conclusion

In this work, we presented a noninvasive, low‐cost optical method based on DRS for accurate measurement of MetHb levels in blood. The presented spectroscopic method supported by ANN allows for continuous monitoring of MetHb concentration. The outcome of this work serves as a promising and important step in the field of monitoring vital signs, particularly MetHb. In this regard, it can be concluded that the hybrid approach adopted in this work by combining a nearly realistic tissue model with effective ANN outperforms the currently available technology for MetHb measurements. This would provide a viable solution for rapid and accurate measurement of MetHb levels, thus improving healthcare quality and reducing the mortality rate in emergency units. More research on human subjects is needed to widely adopt this technique in clinical settings.

## Consent

All the subjects who provided written informed consent to participate have their data utilized for this study.

## Conflicts of Interest

The authors declare no conflicts of interest.

## Data Availability

The data that support the findings of this study are available from the corresponding author upon reasonable request.
